# Effect of Skin Pigmentation and Finger Choice on Accuracy of Oxygen Saturation Measurement in an IoT-Based Pulse Oximeter

**DOI:** 10.3390/s24113301

**Published:** 2024-05-22

**Authors:** Shyqyri Haxha, Chike Nwibor, Mian Ali, Mohamed Sakel, Karen Saunders, Vladimir Dyo, Shakira Nabakooza

**Affiliations:** 1 Department of Electronic Engineering, Royal Holloway, University of London, Egham TW20 0EX, UK; chike.nwibor.2018@live.rhul.ac.uk (C.N.); mian.ali.2019@live.rhul.ac.uk (M.A.); vladimir.dyo@rhul.ac.uk (V.D.); 2 East Kent Hospitals University NHS Foundation Trust, Canterbury CT2 7NT, UK; msakel@nhs.net (M.S.); karen.saunders10@nhs.net (K.S.); 3 Hertfordshire Partnership University NHS Foundation Trust, Hatfield, St. Albans AL3 5TQ, UK; nabakooza.shakira@yahoo.com

**Keywords:** optical sensors, pulse oximetry, Internet of Things, photoplethysmography

## Abstract

Pulse oximeters are widely used in hospitals and homes for measurement of blood oxygen saturation level (SpO_2_) and heart rate (HR). Concern has been raised regarding a possible bias in obtaining pulse oximeter measurements from different fingertips and the potential effect of skin pigmentation (white, brown, and dark). In this study, we obtained 600 SpO_2_ measurements from 20 volunteers using three UK NHS-approved commercial pulse oximeters alongside our custom-developed sensor, and used the Munsell colour system (5YR and 7.5YR cards) to classify the participants’ skin pigmentation into three distinct categories (white, brown, and dark). The statistical analysis using ANOVA post hoc tests (Bonferroni correction), a Bland–Altman plot, and a correlation test were then carried out to determine if there was clinical significance in measuring the SpO_2_ from different fingertips and to highlight if skin pigmentation affects the accuracy of SpO_2_ measurement. The results indicate that although the three commercial pulse oximeters had different means and standard deviations, these differences had no clinical significance.

## 1. Introduction

Pulse oximetry is a non-invasive method of measuring blood oxygen saturation (SpO_2_), which has revolutionised the monitoring of oxygenated and de-oxygenated haemoglobin [1,2]. Due to it being a non-invasive method, it is widely used in all healthcare settings including hospitals and was adopted as the international standard for patient monitoring when administering anaesthesia in early 1990 [3]. Since the COVID-19 pandemic, SpO_2_ has become one of the most important vital parameters of health to monitor clinical status.

It is well known that the accuracy of pulse oximeters is influenced by various factors, such as the fingertip selection, motion artefacts, and light interference [4,5,6]. In particular, the effect of skin colour and finger placement on blood oxygen saturation measurement has attracted significant attention in recent years. Evidence-based research shows that pulse oximeters (POs) may perform less accurately in darker-pigment patients in comparison to patients with lighter pigment or other skin colours [7,8,9,10,11,12]. For example, it was shown that some colours of nail polish (blue, green, and black) absorb more light, hence decreasing the accuracy of POs [8,13,14]. Considering that SpO_2_ level is a vital parameter for diagnosing many conditions, the topic has attracted significant attention recently. Shi et al. [15] investigated the effect of skin pigmentation on pulse oximeter accuracy, and showed that SpO_2_ was overestimated in people with darker skin. Bickler et al. [16] studied the effect of skin pigmentation on pulse oximeter accuracy at low oxygen saturation and showed an overestimated SpO_2_ in darkly pigmented individuals during hypoxia [16]. Adler et al. [17] investigated the effect of skin pigmentation on pulse oximeter accuracy but the results from their work showed that skin pigmentation did not affect the performance of POs. The inaccuracy in oxygen saturation readings has a significant impact, with black patients having more than a 2-fold probability of a falsely high oxygen reading compared to white patients in the ICU [18]. Occult hypoxemia was in turn associated with higher mortality and organ failures [19].

Although there is substantial work on IoT-based pulse oximeters [20,21,22,23], the focus is often on the design and implementation aspects of a particular component, such as communication, calibration, or user application. In particular, Mahgoub et al. [23] concentrate on the design of a health monitoring system with remote alert capability. Chugh et al. [21] focus on a low-cost calibration-free pulse oximeter, while ref. [23] studies the wireless body area network aspect of pulse oximeters. However, the accuracy evaluation of such designs in realistic conditions and their comparison with NHS-approved instruments have not received the required attention.

In this research, we review the physical principles of pulse oximetry and evaluate the performance of our custom pulse oximeter based on an off-the-shelf photodetector and 3D-printed enclosure developed in earlier research [24]. We then compare its performance with that of the three different UK NHS-approved pulse oximeters using extensive measurements and statistical analysis, including the investigation of the impact of skin pigmentation on SpO_2_ measurement accuracy. During the measurements, blood oxygen saturation measurements were taken from 20 healthy volunteers on each finger of both left and right hands, using different sensors. As SpO_2_ is normally measured on the middle or index fingers [25], one of our objectives was to understand how the sensor placement on a particular finger affects the accuracy. A statistical analysis and data visualisation using a Bonferroni test and Bland–Altman plot were used to demonstrate the differences between the devices. Our results indicate that although the commercial pulse oximeters had different means and standard deviations, these differences had no statistical significance.

The rest of the paper is structured as follows. Section 2 describes related work focusing on the impact of skin pigmentation on blood saturation measurement. Section 3 presents the physical principles of pulse oximetry, including the Beer–Lambert law and the construction of pulse oximeters. The coverage of these principles and the subsequent evaluation methodology is geared towards researchers working on IoT systems. Section 4 contains our methodology, including study protocol and recruitment of volunteers, followed by results and conclusions in Section 5 and Section 6, respectively.

## 2. Related Work

Monitoring of blood oxygen saturation (SpO_2_) is vital as a diagnostic of many cardiovascular and respiratory conditions, such as heart attack, chronic obstructive pulmonary disease COPD, asthma, lung cancer, and pneumonia. Maintaining adequate SpO_2_ levels is critical to sustaining life, and SpO_2_ is under constant homeostatic control by the brain, which controls it at a subconscious level. When a person is unwell, the body may not be able to respond adequately and may require medical assistance and intervention. In these circumstances, it is critically important that the SpO_2_ level is accurately measured and external assistance provided to ensure the level does not go below the safe threshold. The normal range for SpO_2_ in humans ranges from 95% up to 100% [26]. When the SpO_2_ level drops below 92%, this is known as ’hypoxemia’ and usually results in observable changes in a person’s physical status including a variety of symptoms, such as shortness of breath, fingernails and lips that can appear blue, rapid breathing, and an increased heart rate. Without prompt medical intervention, a person can become confused, lose the ability to communicate, or lose consciousness and die. Therefore, accurate measurement and timely intervention are critical for ensuring that patients survive.

Pulse oximeters estimate the arterial oxygen saturation (SaO_2_) from the oxygenated and total haemoglobin ratio. Shi et al. [15] investigated the effect of skin pigmentation on pulse oximeter accuracy using 6505 participants over 32 studies with all their datasets obtained from hospital settings. Fifteen of their studies measured skin pigmentation and compared SaO_2_ and pulse oximeter measurements in dark-skinned subjects. The research showed that SpO_2_ was overestimated in people with darker skin [15].

Adler et al. [17] studied the effect of skin pigmentation on pulse oximeter accuracy in an emergency department. The research was carried out in an emergency department section of a hospital, collecting SpO_2_ readings from 284 patients with three skin pigments. The authors determined skin pigmentation through colour swatches with controlled lighting. The arterial blood sample of the volunteers was taken simultaneously as the pulse oximeter measured the SpO_2_ values. The results from their work showed that skin pigmentation did not affect the performance of POs [17].

Bickler et al. [16] showed the effects of skin pigmentation on the pulse oximeter accuracy at low oxygen saturation by evaluating the SaO_2_ level of 11 healthy dark-skin-pigmented subjects. The subjects were placed in a semi-supine position and were allowed to inhale an air mixture of nitrogen, carbon dioxide, and oxygen through a mouthpiece. The method estimated breath-by-breath SaO_2_ using an end-tidal oxygen and carbon dioxide concentration, which was determined by a mass spectrometer. During the measurement, an operator controlled the inspiration of gas to achieve a stable plateau of desaturation. Additionally, three commercially available POs were used to measure the difference between SaO_2_ and SpO_2_. Their findings showed overestimated SaO_2_ in darkly pigmented individuals during hypoxia [16]. For a more comprehensive review of the topic, the reader is referred to the works by Shi et al. [15,27], Al-Halawani [28], and Cabanas [29].

## 3. Principle of Pulse Oximetry

**Figure 1:**
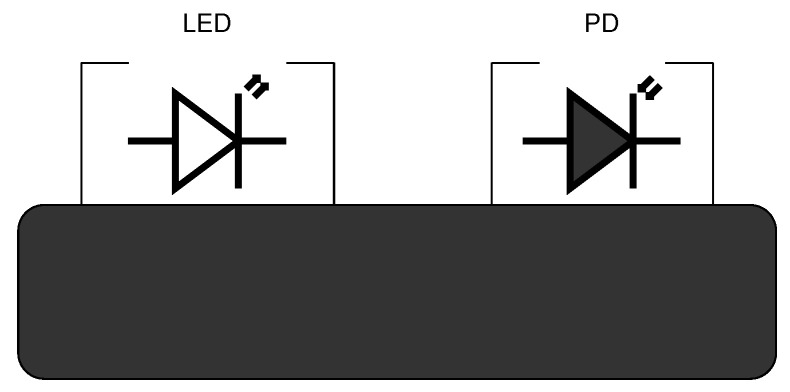
Reflection measurement mode [32].

Pulse oximeters use photoplethysmography (PPG) to detect variations in blood volume within human biological tissues resulting from the heartbeat [30]. PPG is a non-invasive technique that employs a light-emitting source, such as an LED, and a photodetector. The light-emitting source radiates red or infrared light to the skin, and then, measures the amount of light either reflected from the skin surface or transmitted through biological tissue depending on the measurement mode. In reflection mode, the light detected by a photodetector is a result of scattering and reflection from biological tissue. As shown in Figure 1, the LED is placed adjacent to the photodiode within a minimum spacing distance between the diode and skin. The distance should accurately represent the pulsatile PPG component and ranges from 0.5 to 18 mm. There is also separation between the LED and the photodiode to prevent the direct incidence of light from the LED to the photodiode. The precise mounting of this opaque material, fitting the sensor firmly on the skin, and ensuring the area of application is plain and spacious, are important for accurate measurement [31]. The reflective method is convenient for measurement from different parts of the body as both the light source and the photodetector are located close to each other rather than on the opposite sides of the biological tissue, which is the case in transmission mode.

### 3.1. Relationship between Haemoglobin and Absorption of Light

Pulse oximetry is based on the fact that in the red and infrared spectrum, light absorption is different for oxygenated haemoglobin (O_2_Hb) and de-oxygenated haemoglobin (HHb) [2]. Compared to blue, green, yellow, and far infrared wavelengths, which are absorbed by water and vascular tissues, both red and near infrared (NIR) light have deeper penetration depth in biological tissues. The amount of light absorption depends on both haemoglobin type and the wavelength: oxygenated haemoglobin O_2_Hb absorbs more infrared light and less red light, while de-oxygenated haemoglobin HHb absorbs more red light. Consequently, pulse oximeters utilise two wavelengths: NIR at 940 nm and red light at 660 nm [2,33,34]. It should be noted that the light absorption directly depends on the arterial blood volume, which oscillates with the cardiac cycle, rising in systole and descending in diastole, compared to the volume of blood in the capillaries, veins, skin area, fat, and bones, which remains constant.

The light received by the photodetector consists of pulsatile (AC) and stable non-pulsatile components (DC) [2]. The pulsatile component depends not only on the systolic blood pressure (SBP) in the vascular tissue but also on the number of illuminated tissues in the artery [35]. The pulse oximeter uses the peak amplitude of absorbance to evaluate the modulation ratio of red and NIR [2]: $R=\left(A_{red,AC}/A_{red,DC}\right)/\left(A_{IR,AC}/A_{IR,DC}\right)$, where R is the ratio of pulsatile to non-pulsatile components of Red–IR light absorption, and A represents the absorbance. The R-value can be used to evaluate the SpO_2_ level [7]:

(1) $$SaO_{2}=\frac{Oxygenated\;Haemaglobin}{Total\;Haemaglobin}=\frac{HbO_{2}}{HbO_{2}+Hb}$$

**Figure 2:**
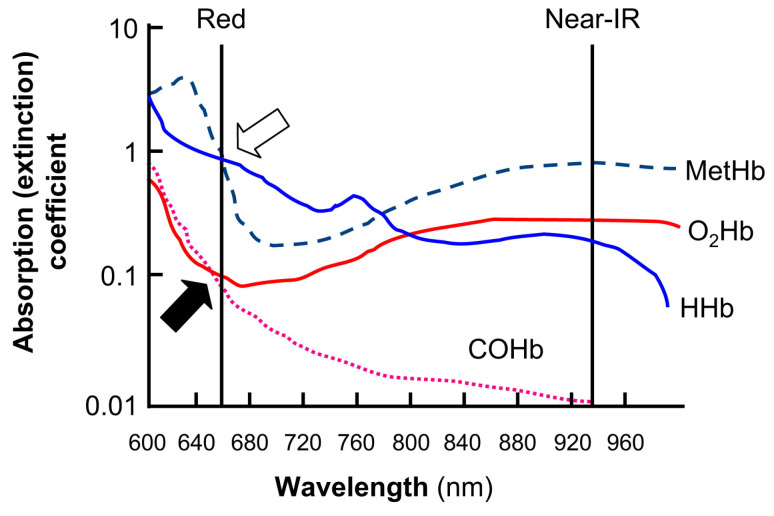
The extinction coefficients for the four different types of haemoglobin and their relationships within red and IR light wavelengths [2].

The Oxygen in the blood is carried by the haemoglobin within red blood cells (RBCs); as the RBCs travel around the body, the oxygen is released by the haemoglobin. The ratio of oxygenated haemoglobin to the total quantity of haemoglobin in the blood is referred to as the oxygen saturation in arterial blood, SaO_2_. It is important to note that other forms of haemoglobin, such as methaemoglobin and carboxyhaemoglobin, affect the accuracy of the pulse oximeter. Methaemoglobin is formed when the iron in the heme group is in the ferric state (${\mathrm{Fe}}^{3+}$) instead of the normal ferrous state (${\mathrm{Fe}}^{2+}$). The methaemoglobin cannot transport oxygen around the body and has a blue-brown pigmentation. Carboxyhaemoglobin develops on inhalation or exposure to carbon monoxide [36,37]. Carbon monoxide has a higher affinity with haemoglobin than oxygen, hence the CO is not released as it becomes attached to the haemoglobin, which reduces the amount of oxygen the blood carries [38] (Figure 2).

The amount of absorbed light is proportional to the Hb concentration in the blood. The relationship between light absorption and Hb is described by the Beer–Lambert law [36], which is the merging of two laws: Beer’s law describes the direct relationship between concentration and absorbance, whereas Lambert’s law describes the direct relationship between absorbance and path length [39]. The Beer–Lambert law is given as [39]

(2) A=ϵlc

where A is the absorbance of light by a sample, l is path length, c is concentration level, ϵ is the molar extinction coefficient, and C is the concentration of the absorbing tissue.

For incident light at a given wavelength and with intensity I_0_ propagating through a concentrated solution c, the intensity of transmitted light can be deduced as

(3) $$I_{i}=I_{0}\times e^{-\epsilon lc}$$

**Figure 3:**
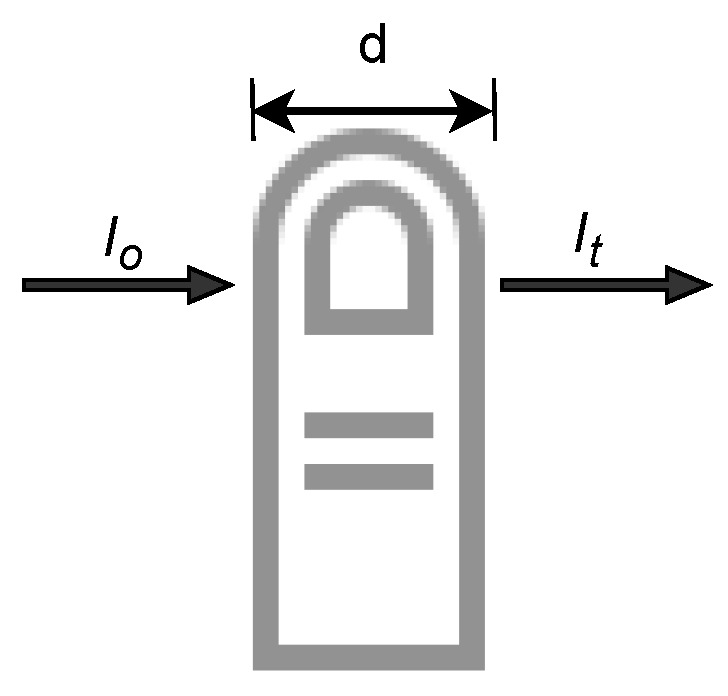
Beer–Lambert model and component [33].

Since we are considering a pulse oximeter, where the measurement area is the fingertip, the fingertip can be regarded as the solution with concentration c. In the transmission of incident light I_i_ at a given wavelength, the light intensity varies, with the change depending on the absorption properties of the fingertip and the path length of the finger, Figure 3 [40,41,42]. To accurately evaluate the total absorbance, a summation of venous and arterial absorbance would be required [2].

(4) $$A_{t}=A_{v}+A_{a}=\epsilon_{v}b_{v}c_{v}+\epsilon_{a}b_{a}c_{a}$$

A PO evaluates absorbance with time, a first-order derivative of the above equation concerning time [2]:

(5) $$dA_{t}/dt=\left(db_{v}/dt\right)\epsilon_{v}c_{v}+\left(db_{a}/dt\right)\epsilon_{a}c_{a}$$

The ratio R of light transmitted to incident light from the red and IR wavelengths of the light source is expressed as

(6) $$R=\frac{log\left(I_{out_{R}}|I_{in_{R}}\right)\lambda_{red}}{log\left(I_{out_{IR}|I_{in_{IR}}}\right)\lambda_{IR}}$$

From the R-value extinction coefficient of haemoglobin, SaO_2_ can be calculated. The mathematical relationship between the extinction coefficient and haemoglobin is denoted as [43]

(7) $$SaO_{2}=\frac{\epsilon_{Hb}\left(\lambda_{1}\right)-R\epsilon_{Hb}\left(\lambda_{2}\right)}{R[\epsilon_{Hb}\left(\lambda_{2}\right)-\epsilon_{Hb}\left(\lambda_{2}\right)]+[\epsilon_{Hb}\left(\lambda_{1}\right)-\epsilon_{HbO2}\left(\lambda_{1}\right)]}\times 100\%$$

where $\epsilon_{Hb}$ is the haemoglobin extinction coefficient, $\epsilon_{HbO_{2}}$ is the extinction coefficient of the oxygenated haemoglobin, and $\lambda_{1}$ and $\lambda_{2}$ are the two separate wavelengths of light [43]. As the value of R decreases the SpO_2_ increases. The peak–peak amplitude of the pulse wave at a high percentage of SpO _2_ is higher in the IR. The low level of SpO_2_ percentage amplitude is higher with red than with infrared. At low SpO_2_, there is an increase in de-oxyhaemoglobin, which absorbs red light better than oxyhaemoglobin.

### 3.2. Empirical Calibration of SpO_2_

As the Beer–Lambert law does not model the scattering of light through the skin surface, a calibration is required. Traditionally, pulse oximeters implement an empirical calibration equation for the estimation of SpO_2_ from derived data from a vast group of healthy normothermic volunteers (non-smokers). This empirical calibration results in a pulse oximeter that depends on a fixed calibration curve to estimate the SpO_2_ level from a PPG-measured signal [44]. Every pulse oximeter manufacturer has a tailor-made calibration curve based on the experiment carried out on their volunteers [45,46]. Some patented algorithms have been obtained, where the R ratio is evaluated from the ratio of the derivatives of the IR and red lights’ intensities, which is then employed in an empirical equation for the computation of the average SpO_2_ percentage [47]. The calibration and validation correct the errors emerging from the Beer–Lambert R-curve model. The comparison curves of the Beer–Lambert law and empirical calibration are available in [48] (shown in fig. 86.4).

Instead of a linear decrease in SpO_2_, the empirical calibration shows a non-linear decrease of 70% in oxygen saturation. At a ratio value of 1.25 R, the data becomes linearised. The Beer–Lambert model only displays oxygen saturation in the range of 30–95%. Empirical calibration makes it possible to represent 100% oxygen saturation. Mapping of SaO_2_ values is performed using the following expression [49]:

(8) $$SaO_{2}=SpO_{2}=\frac{K_{1}-K_{2}\left(Red/IR\right)}{K_{3}-K_{4}\left(Red/IR\right)}$$

where $K_{2}$ is a multiple of the ratio of red to infrared light deflected by the body tissues. The empirically derived coefficient K is related to the different extinctions of Hb and HbO_2_. In practice, the relation between SpO_2_ and R is the same. Equation (3) can be normalised to

(9) $$SpO_{2}=K_{1}+K_{2}R$$

where $K_{1}$ and $K_{2}$ are derived through empirical calibration; in the referenced figure, K_1_ = 110 and K_2_ = −25 [49].

### 3.3. Using RMS Value for Estimation of R-Value

The combination of the RMS AC signal with the mean DC signal can be used to evaluate the R-value for the calibration of SpO_2_ in a pulse oximeter [50]:

(10) $$SpO_{2}=\frac{{\left(AC_{RMS}|DC\right)}_{red}}{{\left(AC_{RMS}|DC\right)}_{ir}}$$

In order to evaluate the AC component, the signal is filtered through a bandpass filter with a bandpass of 0.67–10 Hz. An array of RMS synchronous to the detected peak beat–beat of the rolling RMS signal. The advantage of this method is the elimination of the requirement to find a pure signal from noise. Consequently, the peak is used as a non-pulsatile signal and might display an AC component because of the filter.

## 4. Methods

This research study is conducted to investigate the effect of skin pigmentation and finger placement on pulse oximeter SpO_2_ accuracy.

### 4.1. System Design and Prototype

The blood pressure measurement was obtained non-invasively from participants’ fingertips through a 3D-shaped fingertip casing furnished with an IR LED and photodiode. The PPG signal was obtained using a commercial biosensor ProtoCentral Electronics (Bengaluru, India) ProtoCentral Pulse Express optical PPsensor with MAX30102 and MAX32664D biosensor modules. The optoelectronic unit was set up with red and IR LEDs and a single photodiode, with the connection implemented in reflectance mode. The photodiode has a peak wavelength sensitivity of 950 nm and an angle of intensity of 100. The current I_F_ flowing through the LED or PD is directly proportional to the potential difference V (voltage) and inversely proportional to the resistance R1 (ohms) from the LED or PD [51]: $I_{F}=\frac{V_{P}-V_{F}}{R_{1}}$. The resistor was adjusted to vary the LED brightness level. The amount of light passing through the fingertips varies with the expansion and contraction of the heart, which causes variation in blood volume in the fingertips through the increase and decrease of blood flow. As blood flows through the arteries and capillaries of the fingertips, the PD absorbs less light as some of the light is scattered, which leads to higher resistance of the photodetector. The output produced by the PD has a combination of DC and AC components, where the DC component is not beneficial, while the pulsatile AC component carries important information which is synchronous to the heart rate. The overall architecture of the system was designed to be modular for future scalability in accuracy and efficiency.

**Figure 4:**
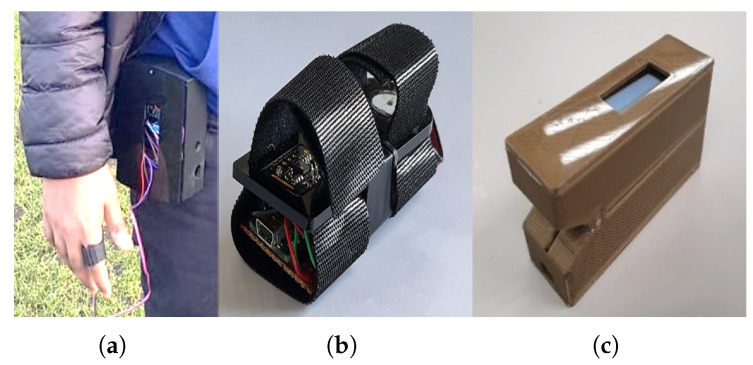
The sensor prototype development timeline [24]. (**a**) Alpha version of the sensor using Velcro tape and bulky 3D case; (**b**) beta version of the sensor; (**c**) the final version of the sensor with 0.91-inch OLED display and remote monitoring capacity.

The sensor was housed in a 3D-printed finger case for better accuracy and for blocking out ambient light. The early version of the 3D case had to be fastened to the waist with a waist strap, while the other part was to be attached to the index finger of the right hand, as shown in Figure 4a. Due to the bulky size of the initial version, a miniaturised version had to be developed, Figure 4b. Further testing revealed that the looseness of the Velcro strap affected the accuracy of the HR measurements in the miniaturised version. This problem was resolved in the final prototype, which had compact dimensions, a 0.91-inch OLED display, and remote monitoring capability, as shown in Figure 4c. The final prototype performed optimally in comparison to the earlier versions. A more detailed description of the sensor design including the communication capability is available in Nwibor et al. [24].

### 4.2. Ethics Approval

The study was conducted by a multi-disciplinary team comprising an electronic engineering team and medical professionals from a large University Teaching Hospital in the UK. The lead researcher obtained ethics approval and Institutional Review Board (IRB) authorisation for conducting this study on neuro-typical healthy volunteers. Reference of the ethics code: REC Project ID: 2882. The study was performed in the laboratory of Microwave Photonics and Sensors at the Department of Electronic Engineering, School of Engineering, Physical and Mathematical Sciences, Royal Holloway, University of London.

### 4.3. Recruitment of Volunteers

We recruited 20 healthy volunteers from (as declared) African, Asian, and Caucasian ethnicity. All our volunteers were between the ages of 25 and 60 years old and were required to be able to communicate (read and write) in the English language, comply with the study procedure, and to be willing to sign our debrief and consent form. All our volunteers were healthy as none of them showed signs of hypertension, hypotension, hypoxemia, bradycardia, or tachycardia. A qualified medical team (physician and nurses) ensured that the correct measurement standards were followed. The demographics of the volunteers are shown in Table 1.

### 4.4. Study Protocol

At the time of taking the SpO_2_ reading from the volunteer, we recorded the skin pigmentation of the volunteer, medical history, lifestyle (smoker or non-smoker), and demographic information alongside the SpO_2_ output from the three commercial PO sensors, as well as our custom-developed sensor.

### 4.5. Measurement Procedure

We made our classified the skin pigment into three distinct categories through the comparison of the skin at the forehead of each volunteer using the Munsell colour system [52]. Our adopted method for assessing skin pigmentation is similar to the method adopted by Adler et al. [17]. This system describes the colour based on 3 axes or scales: hue, chroma, and value. The Munsell colour system is based on a numerical scale with a visually uniform sequence for each colour scale, with each colour having a visual relationship with other colours [53]. The hue axis is used to denote primary colours along the colour spectrum. Chroma denotes the saturated or intensity (weak or strength) of the colour within a colour group, while the value measures how dark or light a colour is. For example, the values of 0 and 10 describe the black and white colours, respectively. The value scale is constant across hue and chroma [17,54].

**Figure 5:**
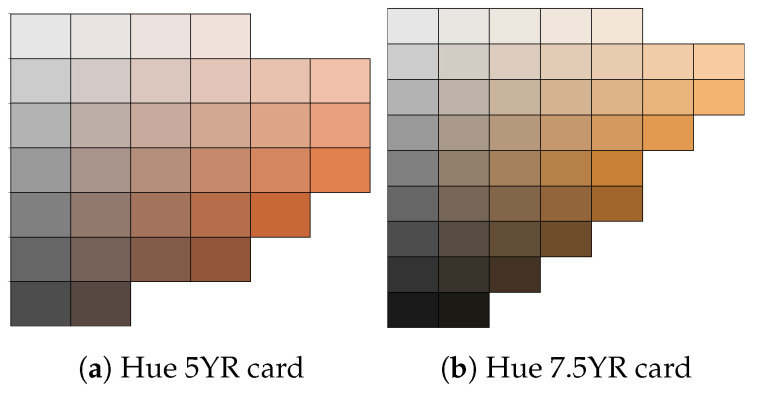
Hue 5YR and 7.5YR hues were used to identify skin colour [54,55].

The Munsell colour system was printed on neutral grey cards with matte tiles mounted on them. We used the Munsell hue 5YR and 7.5YR cards as they closely resemble the human skin colour and provide a range of tiles for various chroma and value ranges. The cards represents a range of colours within the yellow–red hue family. Within this range, there are variations in lightness (value) and chroma, resulting in multiple distinct colours that all fall within the selected hue categories. The volunteers rested for 5 min from their time of arrival as they performed an indirect exercise by walking to the experiment area, and were seated in an upright position with the hand positioned on a table at the same level as the heart. The room was illuminated with a single fluorescent light with an open window for natural light. We compared the skin colour of each volunteer using the 5YR chart shown in Figure 5 and chose the tile closest to the volunteers’ skin pigment. The skin pigments of our volunteers were classified into three categories: dark skin pigment range, with a value of ≤5; brown skin value, with the value within the range 6–7; and white skin pigment, with a value of 8. The chroma colours were classified into 2 categories: category 1 represents skin with darker pigment and falls between chroma levels 1 and 2; while category 2 denotes lighter pigment, with a chroma level ≥ 3.

### 4.6. Finger Placement

**Figure 6:**
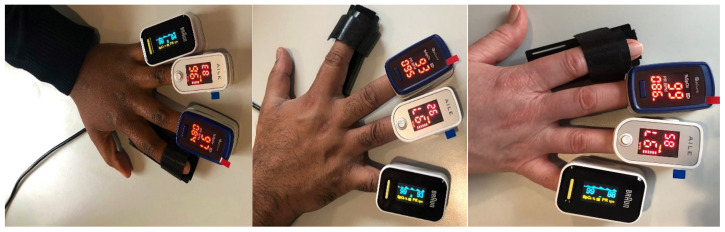
Dark, brown, and white skin pigments with 3 different PO sensors placed on the fingertips of volunteers.

We took a total of 600 SpO_2_ measurements from 20 volunteers, including measurements from each of the 10 different fingertips from both left and right hands (Figure 6). None of the volunteers used nail polish. The measurements were obtained using the UK NHS-approved pulse oximeters Acurio [56], Braun [57], and Ailie [58], which are all fingertip sensors.

Additionally, we took measurements using our custom-developed pulse oximeter and recorded its readings 1 min after obtaining readings from the commercial sensor. After placing the finger in the PO, we waited for 45 s for the reading from the fingertip to stabilise. We then took the highest stable reading from the PO monitor measuring the SpO_2_. The volunteers were asked to rub their hands together to generate a bit of heat in their fingertips to prevent cold hands, which can lead to inaccurate readings.

### 4.7. Statistical Method

For comparing the best finger placement, we analysed the measurements from 10 different fingertips using ANOVA post hoc tests with a Bonferroni correction and an alpha value of 0.05. We also used the Shapiro–Wilk test to assess the normality of the measurements and the Bland–Altman plot to graphically represent the results.

#### Bland–Altman Plot

For comparative analysis and benchmarking of our custom-developed sensor with a commercial BP sensor, we used Bland–Altman plot analysis. This statistical tool assesses bias between measurements conducted by different instruments. In our data analysis, we set the limit of agreement to 95%. On each Bland–Altman plot, the X-axis represents mean values, with each point representing a mean of the two measurements [3,59,60,61]:

(11) (A+B)/2

The Y-axis shows the difference between the measurements, with each point representing a difference between the two measurements:

(12) ([B−A])

There are three important lines in the plot [59]:

1. The average difference between predicted and commercial BP values;
2. The upper limit of agreement, representing the 95% confidence interval for the average difference;
3. The lower limit of agreement, representing the 95% confidence interval for the average difference.

The upper and lower limits are computed based on the mean difference between two datasets and the standard deviation (SD) of that difference [60,61]. The SD of the differences between the custom and commercial sensors provides a suitable index for our comparison. The mean ±1.96 standard deviations (SDs) accounts for the 95% limits of agreement [60,61].

(13) LLoA=Bias−1.96×SD

(14) ULoA=Bias+1.96×SD

where LLoA and ULoA are the lower and upper limits of agreement, respectively.

## 5. Results and Discussion

### 5.1. Determining the Best Finger to Measure SpO_2_

We obtained SpO_2_ values from 10 different fingertips of each volunteer using different pulse oximeters, and then, calculated the mean and standard deviation. The calculated means of the SpO_2_ values from the three pulse oximeters were different for each device. The mapping of finger names to abbreviations is presented in Table 2.

A post hoc Bonferroni test was performed after the ANOVA test was performed to reveal the difference in measurements. We classified the fingers into 10 groups, as shown in Table 2. In each group, we measured 20 SpO_2_ values from each commercial sensor, and then, performed a one-way ANOVA test to investigate if there was any significant difference in SpO_2_ measurements with other fingers. We obtained three different *p*-values from the commercial sensors: Ailie PO, *p*-value = 0.44, F: 0.71; Acurio PO, *p*-value = 0.71, F: 1.00; Braun PO; *p*-value = 0.05, F: 2.76. From these we developed two hypotheses as follows. If *p* < 0.05, there is a significant difference between the means, and *p* ≥ 0.05 shows that there is no significant difference.

The Acurio PO’s lowest standard deviation value was found in R1 (95.80% ±1.85), the Ailie PO’s lowest standard deviation value was found in L4 (98.10% ±0.45), and the Braun PO’s lowest standard deviation value was found in L3 (98.85% ±0.49). We utilised the lowest standard deviation as it shows that the measured values are more spread out, hence why we chose the lowest standard deviation. We performed a post hoc Bonferroni correction test to see which finger groups were different from each other. We also performed an individual *t*-test and controlled the multiple comparisons using Bonferroni correction, i.e., we performed an individual *t*-test for all possible finger combinations.

**Figure 7:**
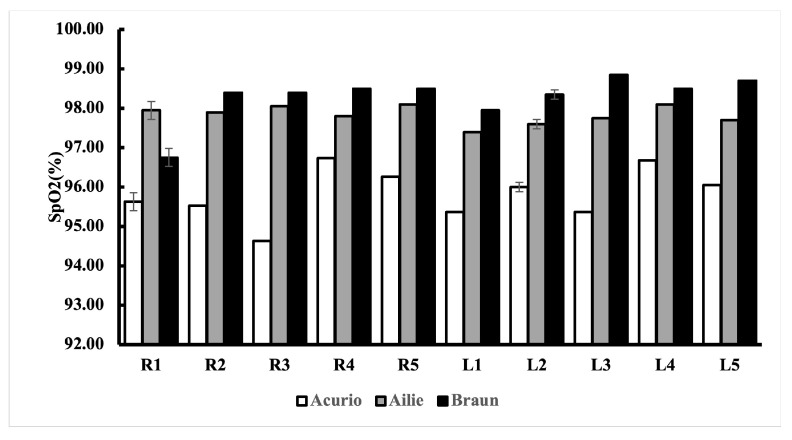
SpO_2_ readings from the 10 different fingertips using different commercial POs, with error bars.

After performing the repeated ANOVA test (Table 3, Table 4 and Table 5), we noticed that there was no significant difference in measuring SpO_2_ from different fingers. Post hoc analyses on the 45 comparisons, showed no significant difference between the commercial sensors (Ailie, Acurio, and Braun), as shown in the tables. We further constructed a bar chart with error bars to show the performance of the 10 fingertips, as shown in Figure 7.

Additionally, Bland–Altman plots were created to show the degree of agreement between the commercial PO sensors and to show if there is clinical significance in the different SpO_2_ measurements between the sensors.

**Figure 8:**
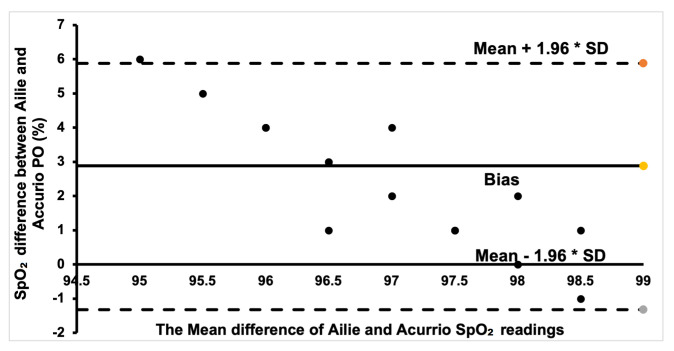
Bland–Altman plot of Ailie and Acurio measurements.

**Figure 9:**
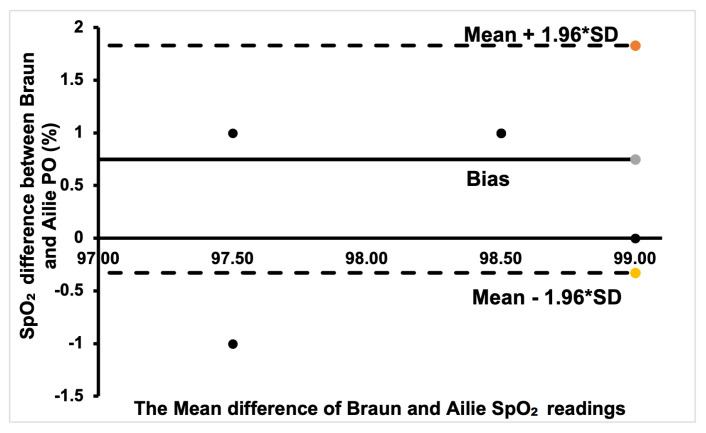
Bland–Altman plot of Braun and Ailie measurements.

The Bland–Altman plot for the Ailie and Acurrio devices in Figure 8 shows a good degree of agreement, with only one outlier value seen outside the limits of agreement. The corresponding data point rallies around the bias point, hence a good degree of agreement is established. Hence, there is no clinically significant difference in the SpO_2_ readings from the two sensors. For the Braun and Ailie POs, the degree of agreement is also strong, as most data points are within the upper and lower limits of agreement, as shown in Figure 9. Hence, there is no clinically significant difference in the SpO_2_ readings between the two sensors.

**Figure 10:**
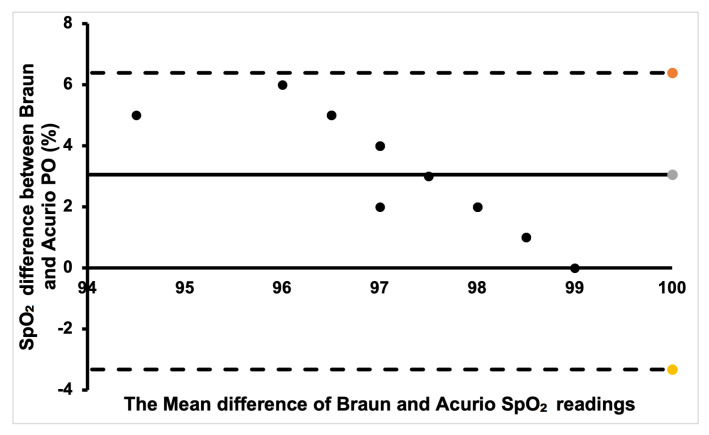
Bland–Altman plot of Braun and Acurio measurements.

Finally, for the Braun and Acurio pair, shown in Figure 10, a good portion of the readings was within the positive bias. A few data points that are far from the bias points denote a weak agreement, with only one point being outside the lower limit of agreement. Therefore, there is no clinical difference in measuring SpO_2_ between the two commercial sensors.

### 5.2. Comparison of Three Different Skin Colours

We classified the skin colours into three categories using the Munsell hue 5YR and 7.5YR cards, as they closely resemble the human skin colour and provide a range of tiles for various chroma and value ranges. We compared SpO_2_ readings from three skin colours, light, brown, and dark, as previously discussed. We carried out a repeated ANOVA test with post hoc analysis on three comparisons using the three different pulse oximeters, white vs. brown, brown vs. dark and dark vs. white, using the best measurement position. We further carried out a post hoc test (Bonferroni test) to further investigate if there was a significant difference between different skin colours. In our hypothesis, a value of *p* ≤ 0.05 indicated a significant difference between the SpO_2_ of the different skin colours. From our analysis, we obtained a *p*-value = 0.79, indicating no significant difference in the measurements and probably no clinical significance. Table 6 summarises the results from the post hoc analysis.

### 5.3. Discussion

In this study, we investigated the relationship between the placement of pulse oximeters on different fingertips and their measurement accuracy. Our study showed that different fingers gave different SpO_2_ values, as shown in Table 3, Table 4 and Table 5, using the ANOVA test (*p* < 0.05) and Bonferroni-corrected test (*p* < 0.001). For the commercial sensors employed, the sensors showed higher SpO_2_ values in some fingers (Ailie = L4, Acurio = R1, Braun = L3). Although there was a difference in readings from the fingers, there was no significant difference in the SpO_2_ values between the fingertips. Additionally, in our study, we showed how three skin pigments (black, white, and brown) affected the SpO_2_ level of our users using different pulse oximeters.

All pulse oximeters performed differently with the fingertips, regardless of the finger measured. The mean and standard deviation in the readings between the fingers differed among the commercial pulse oximeter sensors. The commercial pulse oximeters (Ailie, Acurio, Braun) and our developed sensor did not show a significant difference in measuring from different fingers based on our hypothesis. Post hoc analyses on the 45 comparisons carried out on the fingers showed no significant difference between the commercial sensors and our developed sensor, as shown in the Table 6. Our study was limited to the specific models of pulse oximeters, hence the results might not be applicable to oximeters from different manufacturers.

A survey of healthcare workers showed that 80% of healthcare workers measured SpO_2_ values from the index finger [62]. The authors of the study highlighted the fact that the index finger is fed through the deep palmar arcus, which is formed by the radial artery, whereas the middle finger receives blood from the radial and ulnar arteries. Hence, they concluded that the best-performing fingers for using a PO are the middle and index fingers [62]. The results are largely consistent with previous studies, such as [8,16]. Basaranoglu et al. [63], compared the SpO_2_ values for different fingertips and their results revealed that although there was a difference in SpO_2_ values from the finger, the difference had no clinical importance. These findings corroborate our result which showed a difference in mean measurement from the different fingers and that the difference had no clinical impact on SpO_2_ values. Bickler et al. [16] studied the effect of skin pigmentation on pulse oximeter accuracy at low saturation levels using three pulse oximeters. They observed no significant change in SpO_2_ values at normal SpO_2_ levels but observed a significant change in SpO_2_ levels in darker skin at low SpO_2_ levels (<80%). Their findings corroborate our observation, all our recruited volunteers were healthy with no sign of hypoxemia, hence there was no clinical significance of the effect of skin pigment in our study, as shown in Table 6.

## 6. Conclusions

Since the emergency of COVID-19, SpO_2_ values have become one of the most important vitals to monitor in various clinical areas such as home use, in-patient and out-patient use, hospitals, sports, and other medical uses. In this study, we first provided an overview of the physical principles of SpO_2_ measurement, including the Beer–Lambert law describing the intensity of light propagating through a biological tissue. We then measured SpO_2_ values from 20 volunteers with different skin colours on different fingertips of both hands using three commercially available NHS-approved sensors. We analysed the results statistically using a single-factor ANOVA test and Bonferroni-corrected test to statistically investigate if there was a clinical significance between our custom sensor and the commercial PO sensors. In our pilot study on the effect of skin pigmentation (light, brown, and dark), we concluded that there was no significance in SpO_2_ measurements in healthy individuals with different skin pigments. Through our experiments with our volunteer group, we also concluded that our sensor and three commercial sensors provided different SpO_2_ measurements, although the difference was of no significant importance. Similarly, although the measurements were different for each fingertip, the difference was not statistically significant.

## Figures and Tables

**Table 1:** Demographics of volunteers.

Age	25–60 yrs
Gender (Female/Male)	5/15
Skin:	
White	7
Brown	7
Black	6

**Table 2:** Fingertip abbreviations.

Right thumb	R1	Left thumb	L1
Right index	R2	Left index	L2
Right middle	R3	Left middle	L3
Right ring	R4	Left ring	L4
Right little	R5	Left little	L5

**Table 3:** The Ailie PO’s multiple finger comparison measurements with SpO_2_ mean and SD, using repeated two-way ANOVA test and post hoc test (Bonferroni-corrected at alpha *p* = 0.01).

Finger	SpO_2_ (Mean ± 1.96 × SD)	R1	R2	R3	R4	R5	L1	L2	L3	L4	L5
R1	97.95 ± 0.94	-									
R2	97.90 ± 0.64	NS	-								
R3	98.05 ± 0.69	NS	NS	-							
R4	97.80 ± 1.15	NS	NS	NS	-						
R5	98.10 ± 0.64	NS	NS	NS	NS	-					
L1	97.40 ± 1.90	NS	NS	NS	NS	NS	-				
L2	97.60 ± 1.10	NS	NS	NS	NS	NS	NS	-			
L3	97.75 ± 1.25	NS	NS	NS	NS	NS	NS	NS	-		
L4	98.10 ± 0.45	NS	NS	NS	NS	NS	NS	NS	NS	-	
L5	97.70 ± 0.66	NS	NS	NS	NS	NS	NS	NS	NS	NS	

**Table 4:** The Acurio PO’s multiple finger comparison measurements with SpO_2_ mean and SD, using repeated two-way ANOVA test and post hoc test (Bonferroni-corrected at alpha *p* = 0.01).

Finger	SpO_2_ (Mean ± 1.96 × SD)	R1	R2	R3	R4	R5	L1	L2	L3	L4	L5
R1	95.80 ± 1.85	-									
R2	95.65 ± 2.13	NS	-								
R3	94.8 ± 6.24	NS	NS	-							
R4	96.80 ± 2.25	NS	NS	NS	-						
R5	96.25 ± 3.13	NS	NS	NS	NS	-					
L1	95.35 ± 2.83	NS	NS	NS	NS	NS	-				
L2	95.90 ± 3.73	NS	NS	NS	NS	NS	NS	-			
L3	95.45 ± 3.35	NS	NS	NS	NS	NS	NS	NS	-		
L4	96.70 ± 2.23	NS	NS	NS	NS	NS	NS	NS	NS	-	
L5	96.20 ± 3.16	NS	NS	NS	NS	NS	NS	NS	NS	NS	

**Table 5:** The Braun PO’s multiple finger comparison measurements with SpO_2_ mean and SD, using repeated two-way ANOVA test and post hoc test (Bonferroni-corrected at alpha *p* = 0.01).

Finger	SpO_2_ (Mean ± 1.96 × SD)	R1	R2	R3	R4	R5	L1	L2	L3	L4	L5
R1	96.75 ± 3.63	-									
R2	98.40 ± 0.94	NS	-								
R3	98.40 ± 1.10	NS	NS	-							
R4	98.50 ± 1.19	NS	NS	NS	-						
R5	98.5 ± 1.40	NS	NS	NS	NS	-					
L1	97.95 ± 1.79	NS	NS	NS	NS	NS	-				
L2	98.35 ± 1.27	NS	NS	NS	NS	NS	NS	-			
L3	98.85 ± 0.49	NS	NS	NS	NS	NS	NS	NS	-		
L4	98.50 ± 1.00	NS	NS	NS	NS	NS	NS	NS	NS	-	
L5	98.7 ± 0.73	NS	NS	NS	NS	NS	NS	NS	NS	NS	

**Table 6:** Measurement comparison using repeated two-way ANOVA test and post hoc test (Bonferroni-corrected at alpha *p* = 0.01).

Commercial Oximeter	Skin	SpO_2_ (Mean ± 1.96 × SD)	White	Brown	Black
Ailie	White	98.00 ± 0.53	-		
	Brown	98.14 ± 0.38	NS	-	
	Black	98.14 ± 0.38	NS	NS	-
Acurio	White	96.00 ± 1.35	-		
	Brown	94.00 ± 1.77	NS	-	
	Black	97.00 ± 2.36	NS	NS	
Braun	White	99.00 ± 0.38	-		
	Brown	99.00 ± 0.00	NS	-	
	Black	97.00 ± 0.98	NS	NS	-
Custom	White	99.29 ± 1.11	-		
	Brown	99.86 ± 0.38	NS	-	
	Black	99.29 ± 0.49	NS	NS	-

## Data Availability

Data are contained within the article.
